# Physiology of Stretch-Mediated Hypertrophy and Strength Increases: A Narrative Review

**DOI:** 10.1007/s40279-023-01898-x

**Published:** 2023-08-09

**Authors:** Konstantin Warneke, Lars H. Lohmann, Camila D. Lima, Karsten Hollander, Andreas Konrad, Astrid Zech, Masatoshi Nakamura, Klaus Wirth, Michael Keiner, David G. Behm

**Affiliations:** 1 Institute for Exercise, Sport and Health, Leuphana University, Universitätsallee 1, 21335 Lüneburg, Deutschland Germany; 2 University Sports Center, Carl von Ossietzky University Oldenburg, Oldenburg, Germany; 3 School of Medical and Health Sciences, Edith Cowan University, Joondalup, WA Australia; 4 Institute of Interdisciplinary Exercise Science and Sports Medicine, MSH Medical School Hamburg, Hamburg, Germany; 5 Institute of Human Movement Science, Sport and Health, University of Graz, Graz, Austria; 6 Department of Human Motion Science and Exercise Physiology, Friedrich Schiller University, Jena, Germany; 7 Faculty of Rehabilitation Sciences, Nishi Kyushu University, Ozaki, Kanzaki, Saga Japan; 8 Institute of Sport Science, University of Applied Sciences Wiener Neustadt, Wiener Neustadt, Austria; 9 Department of Sport Science, German University of Health and Sport, Ismaning, Germany; 10 School of Human Kinetics and Recreation, Memorial University of Newfoundland, St. John’s, NL Canada; 11 Institute of Sport Science, Alpen-Adria University Klagenfurt, Klagenfurt, Germany

## Abstract

Increasing muscle strength and cross-sectional area is of crucial importance to improve or maintain physical function in musculoskeletal rehabilitation and sports performance. Decreases in muscular performance are experienced in phases of reduced physical activity or immobilization. These decrements highlight the need for alternative, easily accessible training regimens for a sedentary population to improve rehabilitation and injury prevention routines. Commonly, muscle hypertrophy and strength increases are associated with resistance training, typically performed in a training facility. Mechanical tension, which is usually induced with resistance machines and devices, is known to be an important factor that stimulates the underlying signaling pathways to enhance protein synthesis. Findings from animal studies suggest an alternative means to induce mechanical tension to enhance protein synthesis, and therefore muscle hypertrophy by inducing high-volume stretching. Thus, this narrative review discusses mechanical tension-induced physiological adaptations and their impact on muscle hypertrophy and strength gains. Furthermore, research addressing stretch-induced hypertrophy is critically analyzed. Derived from animal research, the stretching literature exploring the impact of static stretching on morphological and functional adaptations was reviewed and critically discussed. No studies have investigated the underlying physiological mechanisms in humans yet, and thus the underlying mechanisms remain speculative and must be discussed in the light of animal research. However, studies that reported functional and morphological increases in humans commonly used stretching durations of > 30 min per session of the plantar flexors, indicating the importance of high stretching volume, if the aim is to increase muscle mass and maximum strength. Therefore, the practical applicability seems limited to settings without access to resistance training (e.g., in an immobilized state at the start of rehabilitation), as resistance training seems to be more time efficient. Nevertheless, further research is needed to generate evidence in different human populations (athletes, sedentary individuals, and rehabilitation patients) and to quantify stretching intensity.

## Key Points

**Table:** 

Stretching is known to improve flexibility, while resistance training is commonly used to increase strength capacity and hypertrophy.
Both training methods produce mechanical tension, which is a factor known to stimulate anabolic signaling and enhance protein synthesis.
In this review, results extracted from the current literature considering physiological aspects indicate the possibility of using high-volume stretching durations to enhance muscle volume in humans. However, the findings considered are biased by the different methods applied in the literature, particularly in regard to stretching intensity and duration.

## Introduction

In rehabilitation [1, 2] and sports practice [3–6], improving maximal strength and muscle mass is well correlated with joint function and stability as well as sport-specific performance. Most commonly, resistance training is used to increase muscle strength capacity and induce significant hypertrophy [7–11]. In the literature, Goldspink and Harridge [12] refer to muscle force as a reflection of myofilament cross-bridges working in parallel, suggesting a link between fiber cross-sectional area and muscle force [12]. Accordingly, increases in maximal strength via resistance training are often accompanied by hypertrophy [13, 14] due to enhanced muscle cross-sectional area and/or muscle thickness [11, 15, 16]. However, force output also depends on neuromuscular factors such as recruitment, rate coding, and synchronization of related motor neurons [12, 14, 17]. Based on these mechanisms, increased maximal strength induced by resistance training can be assumed to be the result of a wide range of neuromuscular and structural adaptations in response to specific training stimuli.

Periods of immobilization or lower physical activity after serious injury [2, 18, 19] or pandemic lockdowns [20–23] as well as aging in general [24, 25] result in significant loss of muscular performance including atrophy, decrease in maximal strength, loss of flexibility as well as musculoskeletal pain [26]. Morie et al. [27] and Hotta et al. [28] point out the need for safe and efficient alternatives to commonly used training and current therapies in rehabilitation settings to prevent loss of muscle performance and to restore maximal strength and flexibility. While different types of stretch training are known to improve flexibility [29–33], muscle hypertrophy and strength increases are mainly associated with resistance training [15, 34, 35]. However, recent literature demonstrated that, on the one hand, resistance training performed over the full range of motion seems to provide a sufficient stimulus to enhance flexibility [36, 37], while on the other hand, animal studies reported hypertrophy and strength increases by attaching weight/resistance to one wing of a quail or using a stretching apparatus [34, 35] to apply (weighted) stretch to the muscles. Therefore, considering similar effects regarding the main outcomes of strength, muscle mass, and flexibility, shared underlying mechanisms between stretching and strength training could be hypothesized. In humans, there is conflicting evidence regarding stretch-induced muscle hypertrophy and strength gains. Some recently published studies support a potential transferability to human models confirming stretch-mediated hypertrophy and maximal strength increases in humans following long-lasting stretching for up to 2 h per session using calf muscle orthoses or stretching boards [38–41]. These results are opposed by findings showing neither maximal strength increases [42–44], nor hypertrophy [42, 45]. Therefore, the question arises about the underlying mechanisms and the influence of training modalities such as stretching duration or intensity, which could be responsible for the similarities in adaptations between (full range of motion) strength training and (long-duration) static stretching training on muscle hypertrophy.

In strength training, mechanical tension is known to play a key role in morphological and functional adaptations, such as hypertrophy and maximal strength [14, 46, 47], and has also been considered to be one of the most ubiquitous explanatory ﻿processes contributing to stretch-mediated hypertrophy in animals [48–50]. Hypothesizing a potential transferability to humans opens up different opportunities to passively apply sufficient mechanical tension to induce hypertrophy of muscular tissue that counteracts performance and muscle mass losses in rehabilitative settings. However, as no studies were detected exploring the underlying physiological mechanisms of stretch-induced muscle tension and its impact on muscle hypertrophy, this review discusses discrepancies found in the literature in the light of mechanical tension. Furthermore, potential factors contributing to the described heterogeneity in results will be highlighted.

## Impact of Mechanical Tension on Physiological Muscle Adaptations: From the Genotype to the Phenotype

“Form follows function” is a well-known concept propagated by the architect Louis H. Sullivan, which seems applicable to many biological adaptations: specific suprathreshold stimuli cause specific adaptations in the human body. Following the response matrix model developed by Toigo and Boutellier [51], anabolic responses can be traced back to a specific mixture of transcription and translation factors, determined by the type of external stimuli [46, 52, 53]. Inducing mechanical tension is one of the most ubiquitous external stimuli, specifically known from resistance training and seemingly of crucial importance for adaptations of muscular tissue [13, 46]. When performing resistance training, mechanical tension is increased by enhancing the intensity, for example by adding weight to a barbell, leading to enhanced mechanical stress-induced micro-traumatization and exercise-induced muscle damage [54–56]. To induce morphological changes, on a physiological level, gene expression is controlled via external stimuli such as suprathreshold mechanical tension. By applying specific stress, specific signaling pathways are enhanced, transducing external physical stimuli (such as mechanical overload) through biochemical signals to control the corresponding net protein synthesis rate by causing either an anabolic or catabolic milieu [12, 57–59]. Accordingly, Coffey and Hawley [60] described that “[t]he process of converting a mechanical signal generated during contraction to a molecular event that promotes adaptation in a muscle cell involves the upregulation of primary and secondary messengers that initiate a cascade of events that result in activation and/or repression of specific signaling pathways regulating exercise-induced gene expression and protein synthesis/degradation”, which is considered of paramount importance when developing new tissue [61, 62]. It has been shown that several signaling pathways, such as the mammalian target of rapamycin/ribosomal protein/S6 kinases/phosphoinositide 3-kinases (mTOR/p70s6K/PI3K) pathway stimulate anabolic responses for muscle protein synthesis [46, 47, 61, 63, 64] with the highly discussed influence of p70S6K in training-induced hypertrophy [65–68]. Based on this pathway, mechanical overload seems to be responsible for the release of insulin-like growth factor-1 (IGF-1), a key factor in muscle growth [58, 69]. In papers by Tidall [70] and Toigo and Boutellier [51], activation of protein kinase B is described as a response to mechanical overload, which activates the v-Akt murine thymoma viral oncogene/protein kinase-B/mammalian target of rapamycin pathway with a downstream phosphorylation of p70S6K [71, 72] contributing to anabolic responses mainly in two ways: firstly, catabolic or anti-anabolic pathways such as glycogen synthase kinase 3 are reported to be inhibited. Secondly, further growth factors such as myogenin growth factor binding to IGF-related protein (IGFR) activate anabolic kinases by phosphorylation of insulin receptor substrate 1. Hence, a highly complex system of signaling pathways influencing the homeostasis between muscle protein synthesis and degradation is described for resistance training-induced muscle hypertrophy [12, 55–57].

The literature points out alternative ways to induce mechanical tension and therefore mechanical overload on muscular tissue. As early as 1887, Marey [73] demonstrated the adaptability of muscle tissue in animals by surgically moving the triceps surae muscle insertion on the calcaneum farther from the origin leading to an elongation by increasing the distance between the muscle origin and insertion and consequently a stretch, which is reported to result in a significant increase in the serial sarcomere number [74]. Hence, it can be hypothesized that the increased muscle length caused a chronic stretch of the muscle. Accordingly, Alder et al. [75] reported that stretching a muscle increased muscle length, whereas, in contrast, immobilizing a muscle in a shortened position decreased the serial sarcomere number [75, 76]. As the influence of modified innervation patterns (contracting against an attached immobilization device) was discussed in studies investigating the influence of stretching on muscle morphology, Sola et al. [77] applied chronic stretch to denervated bird muscle to exclude the impact of central nervous innervation and was consequently able to attribute the measured hypertrophy effects to passive mechanical tension. In addition to known sarcomere accumulation in series, the authors reported significant hypertrophy effects through parallel accumulation of myofibrils. Even though the healthy comparison group showed (slightly) higher hypertrophic effects, the authors reported significant muscle growth in the denervated muscle. Consequently, the authors hypothesized that skeletal muscle stretch would induce sufficient mechanical tension as the underlying assumed mechanism to induce hypertrophy in parallel [54, 58, 69, 78]. In the following years, stretch-mediated hypertrophy was extensively researched by applying chronic stretching (24 h per day 7 days per week for up to 35 weeks) to one wing of chicken and quail [48–50, 79, 80]. The results are provided and discussed in the following section and Table 1.

**Table 1:** Characteristics of longitudinal animal studies investigating stretch-induced increase in muscle mass in chickens and quails

Study	Animals	Intervention period	(Daily) stretching time and stretch application frequency	Weight added to stretch or stretching apparatus	Results (muscle mass increase)
Alway [88]	Japanese quail (*n* = 20)	30 days	24 h per day, every day	12% of body weight	161.5 ± 7.9%
Alway [99]	Japanese quail (*n* = 70)	30 days	24 h per day, every day	12% of body weight	Young: 45.1 ± 2.1%, aged: 24.1 ± 2.6%
Alway [92]	Japanese quail (*n* = 24)	30 days	24 h per day, every day	12% of body weight	Young: 168.2%, aged: 136.5%
Alway [91]	Japanese quail (*n* = 12)	30 days	24 h per day, every day	12% of body weight	162.5 ± 4.3%
Alway et al. [100]	Japanese quail (*n* = 73)	1–7 days	24 h per day, every day	10% of body weight	6.9 ± 2.7% after 1 day, 21.3 ± 4.7% after 2 days, 41.7 ± 4.7 after 3 days, 44.1 ± 4.4% after 4 days, 59.8 ± 6.5% after 5 days, 58.9 ± 9.6 after 6 days and 64 ± 8.4% after 7 days Muscle mass difference at baseline: 0.5 ± 0.02%
Alway et al. [89]	Japanese quail (*n* = 34)	30 days	24 h per day, every day	10% of body weight	171.8 ± 13.5%
Antonio and Gonyea [101]	Japanese quail (*n* = 18)	16 and 28 days (intermittent stretching protocol)	24 h of stretching on day 1 with 10%, day 4 with 15%, day 8 with 20%, days 11–14 with 25% and days 17–37 with 35% of bodyweight. The days in-between included no intervention (rest)	Progressing from 10 to 35% of body weight	188.1 ± 15.6% after 16 days and 294.3 ± 39.1% after 28 days
Antonio and Gonyea [50]	Japanese quail (*n* = 26)	28 days (intermittent stretching protocol)	24h of stretching on day 1 with 10%, day 4 with 15%, day 8 with 20%, days 11–14 with 25% and days 17–37 with 35% of bodyweight. The days in-between included no intervention (rest)	Progressing from 10 to 35% of body weight	318.6 ± 31.5%
Antonio and Gonyea [82]	Japanese quail (*n* = 7)	15 days (intermittent stretching protocol)	24 h of stretching on day 1, day 4, day 8, day 11, and day 15 The days in-between included no intervention (rest)	10% of body weight	53.1 ± 9%
Ashmore [102]	Chicken (*n* = N/A) [dystrophic]	42 days	24 h per day, every day	Cardboard sleeve	200%
Barnett et al. [79]	Chicken (*n* = N/A)	1, 3, 5, 7, and 10 days	24 h per day, every day	Stretch apparatus	16.9% after 1 day, 35.86% after 3 days, 59.48% after 5 days, 59.74% after 7 days, and 67.35% after 10 days
Bates [86]	Quail (*n* = 16)	35 days	0.5 h, 1 h, 2 h, 4 h, or 8 h per day, respectively, every day	Cardboard sleeve	Daily stretching time of 0.5 h, 1 h, 2 h, 4 h, and 8 h 0.5 h = 57%, 1 h = 60%, 2 h = 67%, 4 h = 72%, 8 h = 150%
Brown et al. [103]	Chicken (*n* = 46)	4, 6, 11, and 16 days	24 h per day, every day	Wing band	Male, 6 weeks old: 66.67% after 6 days Female, 10 months old: 27.12% after 4 days, 32.95% after 11, and 43.23% after 16 days Muscle mass differences at baseline 1.09% Female, 28 months old: 6.06% after 4 days, 12.64% after 11 days, and − 17.65% after 16 days. Muscle mass differences at baseline: 3.51%
Carson and Alway [84]	Japanese quail (*n* = 30)	7 and 14 days	24 h per day, every day	10% of body weight	Young: 98.7 ± 12% after 7 days and 141.6 ± 9.5% after 14 days, aged: 83.9 ± 6.6% after 7 days and 106.9 ± 11% after 14 days
Carson et al. [93]	Japanese quail (*n* = 94)	7 and 14 days	24 h per day, every day	10% of body weight	Adult: 94.1 ± 7.4% after 7 days and 134.7 ± 5.8% after 14 days, aged: 82.1.1 ± 4.9% after 7 days and 102.4 ± 6.2% after 14 days. Muscle mass difference at baseline: 1.8 ± 0.6%
Carson et al. [94]	Japanese quail (*n* = 32)	30 days	24 h per day, every day	10% of body weight	Young: 178.7 ± 7.1%, aged: 142.8 ± 7.9%
Czerwinski et al. [104]	Chicken (*n* = 22)	2 and 11 days	24 h per day, every day	Cardboard sleeve	13% after 2 days and 44% after 11 days
Devol et al. [105]	Chicken (*n* = 101)	5, 10, 15, 20, and 25 days	4 h or 24 h per day, respectively, every day	Cardboard sleeve	Stretching 4 h per day: 8.62% after 5 days, 25.32% after 10 days, 28.84% after 15 days, 41.58% after 20 days, and 32.96% after 25 days Stretching 24 h per day: 35.51% after 5 days, 42.76% after 10 days, 47.55% after 15 days, 41.22% after 20 days, and 33.01% after 25 days
Frankeny et al. [90]	Chicken (*n* = 54)	42 days	2 × 0.25 h, 0.5 h, 1 h, 2 h, 2 × 4 h, 6 h, 8 h or 24 h, respectively, every day	Cardboard sleeve	Daily stretching time varies between 2 × 15 min and 24 h per day. 2 × 0.25 h = 52.9%, 1 × 0.5 h = 70%, 1 × 1 h = 68.8%, 1 × 2 h = 64.41%, 2 × 2 h = 97.83%, 1 × 4 h = 88.46%, 1 × 6 h = 105.77%, 1 × 8 h = 90.45% and 1 × 24 h = 122.43%
Holly et al. [80]	Chicken (*n* = 14)	35 days	24 h per day, every day	Spring-loaded device	63% in PAT muscle, 81% in ALD muscle, and 28% in biceps muscle
Kennedy et al. [83]	Chicken (*n* = 32)	35 days	24 h per day, every day	10% of body weight	100% after 35 days
Laurent and Sparrow [106]	Fowl gallus domesticus (*n* = N/A)	6 days	24 h per day, every day	Attaching a weight (N/A)	74% after 6 days
Lee and Alway [95]	Quail (*n* = N/A)	30 days	24 h per day, every day	12% of body weight	Young: 44.1 ± 3.1%, adult: 32.6 ± 3.9%, old: 25.7 ± 4.3%
Matthews et al. [96]	Japanese quail (*n* = 10)	33 days	24 h per day, every day	10% of body weight	247 ± 91%
Roman and Alway [97]	Japanese quail (*n* = 28)	7, 14, and 21 days	24 h per day, every day	10% of body weight	53.6 ± 2.9% after 7 days, 67 ± 4.4% after 14 days and 70.2 ± 4% after 21 days
Sola et al. [77]	Chicken (*n* = N/A)	14, 28, and 56 days	24 h per day, every day	Attaching a weight of 100 g and 200 g	Peak increase in the ALD with 200-g overload: 158%, 100-g overload: 93% Peak increase in the PLD with 200g overload: 29%, 100g overload: − 4% Peak increase in the TM with 200-g overload: 169%, 100-g overload: 64%
Summers et al. [98]	Chicken (*n* = N/A)	5 days	24 h per day, every day	Cardboard sleeve	Up to 50%
Winchester and Gonyea [107]	Corturnix quail (*n* = 27)	5 and 30 days	24 h per day, every day	10% of body weight	33.6 ± 7.1% after 5 days and 115.3 ± 8% after 30 days

*ALD* anterior latissimus dorsi muscle, *n* number of subjects, *N/A* not available, *PAT* patagialis muscle, *PLD* posterior latissimus dorsi muscle, *TM* teres minor muscle

## Stretch-Mediated Hypertrophy: Results from Animal Studies

If the aim is to answer an important research question, Hooijmans et al. [81] suggested investigating underlying mechanisms in animal studies first. From 1970 to 2000, a vast number of studies using animal models aimed to explore underlying mechanisms of chronic stretch-induced muscle mass increases [77, 82–85]. For that purpose, chickens or quails typically had the muscle of one wing stretched by applying a stretching device [79, 80, 86] or adding weight equivalent to 10–35% of the respective bodyweight to the wing [77, 87–89]. Most of the studies examined the influence of different stretching times that ranged from 2 × 15 min per day (using an intermittent stretching protocol) [86, 90] to 24 h of stretching per day [50, 82, 90–95] on muscle mass, muscle cross-sectional area, fiber cross-sectional area, fiber length and/or fiber number. Some studies investigated physiological adaptations and changes in gene expression and muscle protein synthesis, myosin isoforms [83, 91, 96] as well as myosin heavy chains and myosin light chains [83, 92, 97]. To include a control condition, the contralateral non-stretched muscle was used as an intra-individual control condition [82, 87, 91, 92]. Thus, increases in the intervened muscle were reported under the assumption of no change in the control muscle of the contralateral side.

Most studies investigated the influence of prolonged stretching interventions in the anterior latissimus dorsi, because of its high percentage of slow-twitch fibers, assuming higher responses to chronic stimulation [98]. In 1973, Sola et al. [77] used 100-g and 200-g weights attached to the wing of chickens to induce a stretch stimulus to the anterior latissimus dorsi, posterior latissimus dorsi, and teres minor of one wing leading to an increase in muscle weight of up to 169%. Other authors investigated the influence of mechanical overload via stretching in a flight muscle (patagialis muscle). Muscle mass results for applicable studies are provided in Table 1.

The results in Table 1 show progressive muscle mass growth over time with increases of 53.1% after 7 days to 318.6% after 5 weeks, using daily stretching [87]. In their meta-analysis, Warneke et al. [49] reported increases in animal fiber cross-sectional area of up to 141.6% [89, 93, 94, 99] and an enhanced fiber number of up to 82.2% [82, 84, 87, 89, 93, 94]. These adaptations were accompanied by fiber length increases of up to 50% [87, 89, 99] in chicken and quails. While the literature provides extensive evidence for stretch-mediated hypertrophy in birds, there are only a small number of studies in mammals, showing partially contrasting results.

De Jaeger et al. [100] performed a 4-week stretch training program on rabbit plantar flexors 3 days per week and found significant muscle mass increases of 13.6%. Stretching immobilized muscle, Coutinho et al. [101] found decreases in rat soleus muscle mass following 3 weeks of immobilization of one limb and stretching for 40 min every 3 days during the immobilization phase, while stretching alone without immobilization resulted in no significant changes. Similarly, Gomes et al. [102] determined a significant decrease in muscle mass in rat soleus muscle after 3 weeks of immobilization and stretching for 40 min once weekly. In a subsequent study by Coutinho et al. [103], the authors found that daily stretching for 3 weeks after a prior 4-week immobilization period resulted in a significant increase in rat soleus muscle mass compared with the contralateral control. The methodology used in these studies is therefore not comparable to the procedure used in studies including birds, which are listed in Table 1. Accordingly, lower frequencies and/or stretching durations per session were used, resulting in less hypertrophy, if any. Consequently, the impact of modifying stretching parameters such as duration, frequency, and intensity is more extensively debated in Sect. 5. Apart from differences in load control, a species-dependent difference in adaptations could also be assumed, raising questions about the transferability of stretch-mediated hypertrophy from birds to humans, particularly given presumed differences in the overall protein synthesis rate [49, 104, 105].

## Stretch-Mediated Hypertrophy: Results from Human Studies

As early as 1983, after finding stretch-mediated hypertrophy in animals, Frankeny et al. [90] and Bates [86] requested the investigation of transferability of stretch-induced muscle growth to humans. However, in the only systematic review found on the topic [45], no studies with comparable training parameters could be included. Accordingly, in 2020, the available literature illustrated a lack of muscle hypertrophy with stretching durations of up to 4.5 min per session [106] for up to 24 weeks [107] with a weekly volume of up to 36 min [108]. Most studies with longer stretching durations that found static stretch-mediated hypertrophy were performed after 2020 [38–41, 109–113] and were therefore not included in the aforementioned review [45]. The current state of the literature investigating stretch-mediated hypertrophy is listed in Table 2, with changes in maximal strength listed in Table 3.

**Table 2:** Characteristics of longitudinal human studies on muscle hypertrophy

Study	Subjects	Intervention period (weeks)	Muscle and stretching volume	Methods	Results (hypertrophy increase) mean % increase^a^
Longo et al. [122]	*n* = 30	12	Plantar flexors 2 exercises: each 5 × 45 s/day 5 days/week	Image acquisition: B-mode ultrasound Control condition: separate passive control group	No significant changes
Mizuno [123]	*n* = 35	8	Plantar flexors SS (with additional ES) 4 × 30 s/day 3 days/week	Image acquisition: B-mode ultrasound Control condition: separate passive control group Additional information: 2 IGs, one with SS only and one combining SS with ES	Muscle thickness in SS group + 5.8% Muscle thickness in SS + ES group + 13.4%
Panidi et al. [41]	*n* = 21	12	Plantar flexors 5 days/week starting with 540 s/day and increasing to 900 s/day	Image acquisition: EFOV ultrasound Control condition: contralateral control leg	MCSA in intervened leg + 23% MCSA in control leg + 13%
Sato et al. [42]	*n* = 24	6	Plantar flexors Group 1: 3 × 120 s/day 3 days/week Group 2: 1 × 360 s/day 1 day/week	Image acquisition: B-mode ultrasound Control condition: uncontrolled Additional information: 2 IGs, 1 with 120 s/day and 1 with 360 s/day	No significant changes
Simpson et al. [44]	*n* = 21	6	Plantar flexors 3 min/day 5 days/week	Image acquisition: B-mode ultrasound Control condition: separate passive control group	Muscle thickness + 5.6%
Yahata et al. [38]	*n* = 16	5	Plantar flexors 6 × 5 min/day 2 days/week	Image acquisition: B-mode ultrasound Control condition: contralateral control leg	No significant changes
Warneke et al. [117]	*n* = 52	6	Plantar flexors 60 min/day 7 days/week	Image acquisition: B-mode ultrasound Control condition: separate passive control group and contralateral control leg	Muscle thickness in lateral head of gastrocnemius + 15.3%
Warneke et al. [120]	*n* = 69	6	Plantar flexors 60 min/day 7 days/week	Image acquisition: B-mode ultrasound Control condition: separate passive control group and contralateral control leg	Muscle thickness in lateral head of gastrocnemius + 4.68% Muscle thickness in medial head of gastrocnemius + 7.72%
Warneke et al. [121]	*n* = 45	6	Plantar flexors 60 min/day 7 days/week	Image acquisition: B-mode ultrasound and MRI Control condition: separate passive control group and contralateral control leg	MCSA via MRI in lateral head of gastrocnemius + 8.8% MCSA via MRI in medial head of gastrocnemius + 5.68% Muscle thickness via ultrasound in lateral head of gastrocnemius + 7.9% Muscle thickness via ultrasound in medial head of gastrocnemius + 7.29%
Wohlann et al. [124]	*n* = 44	6	Lower extremity muscles, i.e., quadriceps, hamstrings, gluteal muscles, plantar flexors 4 exercises: each 5 min/day 7 days/week	Image acquisition: B-mode ultrasound in vastus lateralis Control condition: separate passive control group and contralateral control leg	No significant changes

*B-mode* brightness mode, *EFOV* extended field-of-view, *ES* electrical stimulation, *IGs* intervention groups, *min* minutes, *MCSA* muscle cross-sectional area, *MRI* magnetic resonance imaging,* n* number of participants, *sec* seconds, *SS* static stretching

^a^Because of inconsistency in reporting, the % change is only stated as the mean value

**Table 3:** Characteristics of longitudinal human studies on maximal strength

Study	Subjects	Intervention period	Muscle and stretching volume	Methods	Results (maximal strength increase) mean % change^a^
Abdel-Aziem and Mohammad [125]	*n* = 75	6 weeks	Plantar flexors 5 × 30 s twice/day 5 days/week	Contraction type: dynamic (concentric and eccentric) Control condition: separate passive control group Additional information: 1 IG with trained participants, 1 IG with untrained participants	Trained participants: concentric peak torque 30°/sec + 9.11%, concentric peak torque 120°/sec + 11.64%, eccentric peak torque 30°/sec + 11.92%, eccentric peak torque 120°/sec + 12.54% Untrained participants: concentric peak torque 30°/sec – 0.25%, concentric peak torque 120°/sec + 14.51, eccentric peak torque 30°/sec + 11.96%, eccentric peak torque 120°/sec + 9.38%
Akagi and Takahashi [116]	*n* = 19	5 weeks	Plantar flexors 3 × 2 min/day on 6 days/week	Contraction type: isometric Control condition: contralateral control leg	No significant changes
Caldwell et al. [126]	*n* = 30	2 weeks	Quadriceps and hamstrings 3 × 30 s, either once or twice per day for 14 days	Contraction type: isometric Control condition: contralateral control leg Additional information: 2 IGs, 1 IG stretched once/day, 1 IG stretched twice/day	IG with 2 sessions/day + 7.1% in quadricep MVC No significant increase for both groups in hamstring MVC and quadricep MVC for IG with 1 session/day
Chen et al. [127]	*n* = 30	8 weeks	Hamstrings 30 × 30 s/day 3 days/week	Contraction type: isokinetic concentric at 60°/sec Control condition: separate passive control group Additional information: MVC tested in quadriceps as well	MVC in flexion (hamstrings) + 8.67% MVC in extension (quadriceps) + 3.04%
Guissard and Duchateau [128]	*n* = 12	6 weeks	Plantar flexors 10 min/day 5 times/week	Contraction type: ballistic and isometric Control condition: contralateral control leg	No significant changes
Ikeda and Ryushi [129]	*n* = 25	6 weeks	Knee extensors 6 × 30 s/day 3 days/week	Contraction type: isometric Control condition: separate passive control group	MVC + 10.16%
Kokkonen et al. [130]	*n* = 38	10 weeks	Lower extremity muscles, i.e., quadriceps, hamstrings, gluteus, abductors, adductors, plantar flexors, dorsiflexors 15 exercises: each 3 × 15 s/day 3 days/week	Contraction type: dynamic 1RM Control condition: separate passive control group	Knee flexion 1RM + 15.3% Knee extension 1RM + 32.4%
Konrad and Tilp [131]	*n* = 41	6 weeks	Plantar flexors 4 × 30 s/day 5 days/week	Contraction type: isometric Control condition: separate passive control group and contralateral control leg	No significant changes
LaRoche et al. [132]	*n* = 29	4 weeks	Hip extensors 10 × 30 s/day 3 days/week	Contraction type: isokinetic hip extension at 60°/sec Control condition: separate passive control group	Peak torque + 5.3%
Longo et al. [122]	*n* = 30	12 weeks	Plantar flexors 2 exercises: each 5 × 45 s/day 5 days/week	Contraction type: isometric Control condition: separate passive control group	No significant changes
Marshall et al. [133]	*n* = 22	4 weeks	Hamstrings 4 exercises: each 3 × 30 s/day 5 days/week	Contraction type: isokinetic at 30°/sec and 120°/sec Control condition: separate passive control group	No significant changes
Mizuno [123]	*n* = 35	8 weeks	Plantar flexors 4 × 30 s/day 3 days/week	Contraction type: dynamic 1RM Control condition: separate passive control group Additional information: 2 IGs, 1 with SS only and 1 combining SS with ES	SS + 20.8% SS&ES + 22.4%
Nakamura et al. [43]	*n* = 40	4 weeks	Plantar flexors 3 × 60 s/day 3 days/week	Contraction type: isometric Control condition: separate passive control group Additional information: high-intensity and low-intensity static stretching groups	No significant changes
Nakao et al. [134]	*n* = 30	4 weeks	Hamstrings 1 × 5 min/day 3 days/week	Contraction type: isometric and isokinetic at 60°/sec and 180°/sec Control condition: separate passive control group	Peak torque 60°/sec + 10.55% Peak torque 180°/sec + 13.61% No significant changes in isometric testing
Nelson et al. [130]	*n* = 25	10 weeks	Plantar flexors 4 × 30 s/day 3 days/week	Contraction type: dynamic 1RM Control condition: separate passive control group and contralateral control leg	1RM in intervened leg + 29% 1RM in control leg + 11%
Sato et al. [42]	*n* = 24	6 weeks	Plantar flexors Group 1: 120 s/day 3 days/week Group 2: 360 s/day 1 day/week	Contraction type: isometric at 30° plantar flexion, neutral and 15° dorsiflexion Control condition: uncontrolled Additional information: 2 IGs, 1 with 120 s/day 3 days/week and 1 with 360 s/day 1 day/week	No significant changes
Simpson et al. [44]	*n* = 21	6 weeks	Plantar flexor 3 min/day 5 days/week	Contraction type: isometric Control condition: separate passive control group	No significant changes
Warneke et al. [117]	*n* = 52	6 weeks	Plantar flexors 60 min/day 7 days/week	Contraction type: dynamic 1RM and isometric Control condition: separate passive control group and contralateral control leg	MVC + 16.2% 1RM + 25.3%
Warneke et al. [120]	*n* = 69	6 weeks	Plantar flexors 60 min/day 7 days/week	Contraction type: isometric with extended and bent knee joint Control condition: separate passive control group	MVC with extended knee joint + 18% MVC with bent knee joint + 9.58%
Warneke et al. [121]	*n* = 45	6 weeks	Plantar flexors 60 min/day 7 days/week	Contraction type: isometric with extended sand bent knee joint Control condition: separate passive control group and contralateral control leg	MVC with extended knee joint + 9.44% MVC with bent knee joint + 4.84%
Warneke et al. [40]	*n* = 70	6 weeks	Plantar flexors 60 min/day or 120 min each 7 days/week	Contraction type: isometric Control condition: separate passive control group and contralateral control leg Additional information: 2 IGs, 1 IG stretched 60 min/day, 1 IG stretched 120 min/day	MVC in 60-min group + 15.3% MVC in 120-min group + 22.9%
Warneke et al. [118]	*n* = 35	6 weeks	Plantar flexors 10 min/day 7 days/week	Contraction type: isometric Control condition: separate passive control group	MVC + 10.5%
Wohlann et al. [124]	*n* = 44	6 weeks	Lower extremity muscles, i.e., quadriceps, hamstrings, gluteus, plantar flexors 4 exercises: each 5 min/day 7 days/week	Contraction type: isometric in leg press Control condition: separate passive control group and contralateral control leg	MVC + 4.4%
Worrell et al. [135]	*n* = 19	3 weeks	Hamstrings 4 × 15–20 s/day 5 days/week	Contraction type: isokinetic at 60°/sec and 120°/sec Control condition: uncontrolled	Eccentric peak torque 60°/sec + 8.5%, eccentric peak torque 120°/sec + 13.5%, concentric peak torque 120°/sec + 11.2%, no significant increase for concentric peak torque 60°/sec
Yahata et al. [38]	*n* = 16	5 weeks	Plantar flexors 6 × 5 min/day 2 days/week	Contraction type: isokinetic at 30°/sec and 120°/sec and isometric at 30° plantar flexion and neutral Control condition: contralateral control leg	MVC isometric with neutral ankle angle + 6.4 MVC isokinetic at 120°/sec + 7.8% Other tests showed no significant changes

*1RM* one repetition maximum, *ES* electrical stimulation, *IG* Intervention group, *min* minutes, *MVC* maximal voluntary contraction, *MVIC* maximal voluntary isometric contraction, *n* number of participants, *sec* seconds

^a^Because of inconsistency in reporting, the % change is only stated as the mean value

The animal research on birds in Table 1 shows consistent effects regarding stretch-induced hypertrophy using different stretching techniques such as attaching weight to the wing and using cardboard sleeves or stretching bands to apply stretching times that ranged from 0.5 h to 24 h per day every day (except for the three studies by Antonio and Gonyea [50, 82, 114]) for up to 56 days. Conversely, Tables 2 and 3 illustrate conflicting results in human studies regarding stretch-induced hypertrophy and strength using stretching devices such as stretching boards or orthoses, while only Simpson et al. [44] used external weights via a leg press to perform 3 min of stretch per session. While hypertrophy and strength increases can be found with stretching times of as little as 4 × 30 s per day, 3 days per week for 8 weeks [115], other interventions with similar or longer stretching durations per session and overall intervention periods reported no significant changes [38, 42, 116]. Studies using a comparable load control to animal studies were performed in 2022 and 2023 applying high stretching volumes of 7–14 h per week with daily stretching of 1–2 h [40, 109, 112, 113] showing more consistent effects regarding hypertrophy and strength increases.

## Discussion

The aim of this narrative review was to discuss the impact of stretch-induced mechanical tension as an underlying mechanism on muscle hypertrophy and strength enhancements.

### Stretching-Induced Muscle Hypertrophy and Maximal Strength in Animals

There are different approaches to explain stretch-mediated hypertrophy. Most prominently, the majority of the authors from animal studies attributed increases in muscle mass to stretching-induced mechanical overload [77, 90]. Devol et al. [117] referred to mechanical tension per sarcomere as an important factor inducing hypertrophy, which would explain the rapid increase in fiber cross-sectional area in the first days [97, 99]. When the stretch stimulus was not re-adjusted, hypertrophy plateaued after these first few days [79, 97], which was suggested to be the result of longitudinal hypertrophy (increased number sarcomeres in series) causing a reduction of mechanical tension per sarcomere. Accordingly, supporting the initially stated hypothesis of shared mechanisms between strength and stretch training, Ashmore [118] indicated that “[t]he common factor present in all cases of muscle growth is that tension on the myofibrils is present. The tension may be actively or passively conveyed to the contractile proteins. It seems likely that rate of muscle growth is proportional to the time that tension is applied to the muscle fiber.” Furthermore, similar stretching-induced signaling pathways, biochemical, and physical changes [80, 118–122] including growth hormone and IGF-1 [117, 123] were frequently reported in animal experiments, supporting the importance of stretching-induced mechanical overload to activate anabolic pathways possibly via stretch-activated channels [72, 124, 125]. Increases in muscle mass were accompanied by an increase in deoxyribonucleic acid and ribonucleic acid levels [79, 126], growth promoting factors [61, 98, 127] and IGF-1 messenger ribonucleic acid [128] as well as increases in the rates of muscle protein synthesis [61, 119, 126, 129]. Interestingly, comparable with strength training, Sasai et al. [69] demonstrated that mTOR/p70S6K is activated acutely after stretching. Furthermore, Agata et al. [130] showed significantly increased phosphorylation of Akt and p70S6K due to a stretching intervention period of 2 weeks in which the rat soleus was stretched with an intermittent protocol for 15 min per day.

In the literature on birds, further specific adaptations following stretching interventions were reported. Roman and Alway [97], Alway [88] and Kennedy et al. [83] showed a shift in myosin heavy chain expression toward slower myosin isoform of slow myosin 2, decreasing contraction velocity [118], which was explained as a more favorable energetic state of the muscle to handle chronic mechanical overload [96]. Furthermore, an increase in fiber number was reported frequently [48, 77, 82, 114] and may be attributable to hyperplasia effects. The authors discussed different theories including splitting of existing muscle fibers and activation of satellite cells [50, 77, 85, 96]. Just one study by Alway [91] determined an approximately 95% maximal strength increase following 30 days of chronic stretch. Finally, Sola et al. [77] concluded that “[i]f stretch can be maintained, there appears to be little limit to extent and duration of the hypertrophy” (p. 95). Based on these studies, the transferability to mammals remains speculative, as there were substantial differences from the studies on birds [100–103].

There are different limitations, complicating a conclusive final statement attributing adaptations exclusively to mechanical tension. Chicken and quail stretched one side only, partially using weights attached to the wing. Thus, it is difficult to distinguish between passive mechanical overload and contraction of the bird against the weight causing muscle mass increases [89]. However, consideration of studies from Holly et al. [80] and Barnett [79] describing no increased electromyographic activity in the stretched muscles and Sola et al. [77] reporting reduced but still significant stretch-induced hypertrophy in denervated muscle, indicates that voluntary contractions against the stretching device may not be a pre-condition for hypertrophy. While Hotta et al. [28] pointed out a significantly reduced blood flow to the stretched muscle, further explanatory approaches, such as hypoxia, however, were not discussed in most animal studies. Notably, in humans, muscle blood flow restriction training has shown hypertrophic responses [131–134]. Additionally, because animals had to be dissected for examination, there was no real control group included in any of the listed studies, lowering the study quality. Finally, the recently performed meta-analysis [49] shows significant limitations, as high study heterogeneity as well as dependency of effects were ignored. Although high consistency in the direction of effects can be assumed (almost all showed increases), multiple effect sizes originating from the same study were not pooled, leading to an overestimation of the concluded effect size. In summary, while in mammals results are contradictory, high consistency in chronic stretch-induced muscle hypertrophy in birds was reported. The explanatory approach including mechanical tension as the only stimulus seems to be limited and one-dimensional.

### Stretching-Induced Muscle Hypertrophy and Maximal Strength in Humans

While animal studies exhibit high effect consistency, there are contrasting results in humans raising questions about the transferability of animal results [49, 86, 90]. Previous reviews by Shrier [135], Medeiros and Lima [136] and most recently Arntz et al. [137] reported small magnitude effects of stretch training on maximal strength; however, no systematic review found stretch-induced hypertrophy in humans. Accordingly, in 2020, Nunes et al. [45] were not able to demonstrate stretch-induced hypertrophy. Of note, in contrast to animal research, most human studies used short stretching durations showing no significant hypertrophy. Only Simpson et al. [44] and Mizuno et al. [115] reported muscle thickness increases of 5.6% using 3 min of plantar flexor stretching 5 times per week for 6 weeks and 5.8% following 8 weeks of plantar flexor stretching 2 min per session with 3 sessions per week, respectively. However, the statistical procedure in the Simpson et al. [44] study was criticized, with a request for a recalculation of the results [138]. Since 2020, new evidence emerged examining longer stretching durations and/or higher training frequencies. Yahata et al. [38] used session durations of 30 min (divided into 6 × 5 min performed 2 days per week) and reported significant maximal strength increases without accompanied hypertrophy. Panidi et al. [41] observed plantar flexors muscle hypertrophy of 23% in the intervened leg and approximately 13% in the control leg using stretching durations of up to 15 min, 5 days per week in adolescent female volleyball players in addition to regular volleyball training. Of note, no passive control group was included in either study. In 2022 and 2023, Warneke et al. [40, 109, 112, 113] examined the effects of daily 1-h stretch training on muscle growth and strength parameters, reporting significant hypertrophy. With enhancements in muscle thickness of approximately 5–15%, the results seem to be comparable to those reported from resistance training, with hypertrophy of up to 15% following 12 weeks of resistance training [139–141], but limited to the calf muscles. Consequently, performing long-lasting stretching could be hypothesized as an alternative when aiming to induce muscle hypertrophy. Interestingly, the effects of 1 h of daily stretching were directly compared to a 3-days per week 5 × 12 repetition calf raise training, with no significant difference in adaptations regarding maximum strength, hypertrophy, and flexibility observed. In a randomized trial in humans, there was an 18% gain in plantar flexor strength in full knee extension following stretching and a 13% gain in strength following a 5 × 12 repetition calf raise training 3 times a week with an extended knee joint. When the knee was bent to 90 degrees for isometric testing, the results suggested a ~ 10% increase for both conditions [112]. Although the participants were described as “recreationally trained”, it can be assumed that the participants were not familiar with long-lasting stretching, but were likely familiar with strength training. Therefore, the unexpectedly high stretch-induced increases could possibly be attributed to a new training stimulus. Because exclusively long-lasting static stretching studies were able to show hypertrophy, the practical applicability seems to be limited to special circumstances. For example, it would be difficult to practically implement and compare 7 h of calf stretch training to 3 × 15 min of calf raise training per week [142]. Furthermore, almost all studies reporting increases in maximal strength or hypertrophy used external devices, either a stretching board [38, 41, 110] or stretching orthoses [109, 112, 113] and applied long-lasting stretching to isolated, lower extremity muscle groups, typically the plantar flexors [38, 41, 44, 109, 112, 113] or the hamstrings [143]. Aiming to transfer measured adaptations to larger muscle groups and more complex movements, Wohlann et al. [116] applied 4 static stretches to the lower extremity, including the plantar flexors, hamstrings, quadriceps, and gluteal muscles (each for 5 min daily) without using stretching devices. Even though maximal strength increased slightly (by about 4%), no muscle hypertrophy was found.

There are different explanatory approaches for the discrepancies in the results. Firstly and most likely, following the theory of mechanically-induced protein synthesis stimulation, stretching intensity can be assumed to play a key role in adaptations [144]. In line with this theory, van der Pjil et al. [145, 146] described the importance of titin unfolding for stimulating anabolic signaling leading to hypertrophic responses. As titin can be assumed to unfold with high sarcomere lengths only, reaching high muscle lengths seems essential, assuming the activation of titin kinase involving downstream pathways [46]. Furthermore, Wackerhage et al. [46] described titin unfolding related interactions with protein synthesis influencing factors such as muscle RING-finger protein 1 and 2—proteasome and autophagy signaling, which are hypothesized to be involved in muscle protein synthesis regulation. Apart from titin unfolding, mechanically overloading filamin is described to be associated with further biochemical responses known to be linked to hypertrophy, such as the Chaperon regulator-3, mammalian target of rapamycin complex-1 (Bag3/mTORC1/YAP) [transcriptional co-activator] pathway [46]. If quantified, most studies used a subjective measure of pain threshold to determine the stretching intensity. Unfortunately, however, in a cross-sectional study, Lim and Park [147] found no correlation between passive torque (passive tension of the muscle developed versus the stretching) and subjective pain perception. When assuming mechanical tension (measured via passive torque) to be of high importance for muscle hypertrophy induced via stretching, a more objective intensity documentation by quantifying passive torque could be an appropriate option. For example, stretching intensity could be stated as a percentage value of the measured maximal passive torque to improve objectivity—comparable with strength training stating the percentage of the one repetition maximum. However, as no studies could be found using this procedure, the practical applicability remains unclear.

Further heterogeneity is observed with intervention periods. In 2018, Freitas et al. [148] reviewed the available literature and concluded that it seems unreasonable to assume structural adaptations following about 20 min of stretching per week for less than 8 weeks. Consequently, no hypertrophic adaptations could be assumed in most of the listed studies using intervention periods of less than 6 weeks and/or weekly stretching volumes of below 20 min [44, 45, 115, 149, 150]. In contrast, considering underlying physiological mechanisms as well as animal research, using high volumes of stretching as evaluated by Warneke et al. [112, 113], Wohlann et al. [116], Panidi et al. [41] and Yahata et al. [38] seems logical.

It is well known that there are further factors influencing training adaptations. Almost all of the studies included untrained or recreationally active participants [38, 42–44, 108, 143, 151–156]. In another study, participants were described as “trained” when performing aerobic activity 3 times per week for more than 20 min per session [157]; however, this categorization of “highly trained” may be debatable. Consequently, studies showing stretch-mediated hypertrophy in well-trained participants or even elite athletes are lacking. Therefore, maximal strength increases of up to 29% using 4 × 30 s of stretch 3 times per week [151] or 23% of hypertrophy [41] must be considered critically, since those increases were higher than or similar to reported adaptations from strength training [7], which should still be considered the gold standard when maximizing strength and muscle mass.

Unexpectedly high hypertrophy effects could possibly be attributed to limitations of the measurement devices. Stretch-mediated hypertrophy was measured via ultrasound in most of the listed studies [41, 49, 112, 116, 153]. Even though ultrasound is stated as highly reliable and valid [158], English et al. [159] as well as Hebert et al. [160] raised concerns about using sonography to assess muscle thickness, referring to low quality and emphasizing the dependency of the measured muscle thickness on the investigator-dependent pressure applied on the transducer. Accordingly, magnetic resonance imaging is described as the gold standard measurement investigating hypertrophy [161], which was exclusively used in Warneke et al. [113]. Further heterogeneity in study design arises from potential sex-related differences. Panidi et al. [41] assessed female participants only, while most authors performed stretching with male participants [38, 42–44, 108, 143, 157]. Assuming differences in hormonal status influence muscle hypertrophy [162], only Warneke et al. [111] explored sex-related differences in stretch-mediated hypertrophy and maximal strength increases showing significantly higher effects in male participants. In accordance with the present explanatory approach, the authors hypothesized that lower flexibility baseline values in male individuals would enhance the applied absolute mechanical tension with stretching compared to female individuals and therefore enhance the magnitude of adaptations.

Apart from mechanical tension-induced signaling cascades, other explanations such as blood flow restriction [28, 131, 133] or neuromuscular adaptations [109, 151] should be considered in further studies. Literature exploring the underlying physiological mechanisms in humans is scarce. In accordance with hypotheses from animal studies, Kremer [72] described the activation of growth factors such as fibroblast growth factor, IGF-1, and mTOR leading to stimulation of anabolic pathways such as Akt/PKB, tuberous sclerosis 1 and 2 due to stretch training via stretch-activated channels [69, 125] linked to further physiological responses [68]. It is hypothesized that there are contractile and metabolic adaptations due to changes in protein synthesis via changes in protein kinases and transcription factors after mechanically overloading the muscle, independent of resistance or stretch training [124]. Only Fowles et al. [56] investigated the acute effects of 33 min of stretching on protein synthesis and failed to show any changes. Furthermore, Smith et al. [163] reported stretching to be sufficient to induce micro-traumatization of the muscular tissue in the long term. However, Wohlann et al. [116] were not able to reproduce these results using 4 exercises, performed for 5 min per day. Moreover, the authors described the changes in creatine kinase values reported by Smith et al. [163] as clinically irrelevant. Therefore, no comprehensive conclusions because of high heterogeneity in the study design as well as the outcomes can be drawn.

### Effects of Long-Term Stretching on Longitudinal Hypertrophy

Increases in muscle mass could also be attributed to increases in muscle fascicle length by increases in serial sarcomere number. Currently, there is only indirect evidence for longitudinal hypertrophy in humans, derived from theoretical or simulated muscle models [164, 165]. However, there are some indications for enhanced muscle lengths. Chen and colleagues and LaRoche et al. [143, 156] reported beneficial effects on force development, while Yahata et al. [38] showed improved maximal strength production in long muscle lengths, while no adaptations were reported in short muscle lengths after stretching interventions. As stated by Kruse et al., “[…] it is assumed that stretching treatments may also induce such adaptations in humans […]. However, the scientific evidence has not yet confirmed this assumption and the overall effectiveness of stretching in humans is still in question […].”[165].

### Limitations

As this is a narrative review, the authors attempted to reflect the essential state of the literature by performing an extended study search. However, because there is a vast number of studies, especially regarding the effects of stretching on flexibility in humans and on hypertrophy in animals, it was necessary to focus the literature search, which possibly led to some studies missing in the review article. To analyze studies addressing our research question, we started by screening recent systematic review articles addressing the topic [45, 48, 49, 135, 136]. Subsequently, related articles and reference lists were screened to find articles excluded in the aforementioned systematic reviews. Furthermore, only studies investigating the effects of stretching on strength or strength-related parameters, such as peak torque, maximal voluntary contractions (eccentric, isometric, or concentric), or muscle mass-related parameters, were considered in this review. For a comprehensive review of the literature, systematic reviews are needed, focusing on the effects of stretching on different outcomes—flexibility, maximal strength and muscle hypertrophy—separately. Obviously, there are fundamental differences regarding strength training and stretching. Although there seem to be similarities in the outcomes (hypertrophy, maximal strength) induced by (high-volume) stretching or strength training, a narrative review does not provide mean effect sizes or confidence intervals. However, based on the studies discussed above, it seems reasonable to conclude muscle hypertrophy may occur using long-lasting stretching interventions (> 30 min per muscle per session) in humans as well as in animals. Unfortunately, because no study explored signaling pathways or muscle protein synthesis rate, the underlying mechanisms can only be discussed based on animal research, and therefore remain speculative in humans.

### Practical Applications

Practical applicability is limited assuming that commonly used resistance training would result in similar adaptations with significantly less time effort compared with long-duration stretch training [112]. However, in situations without access or the possibility to perform strength training, the possibility of stretch-induced muscle hypertrophy should be kept in mind especially in situations of reduced physical activity or prolonged phases of immobilization after injury or surgery. Because no active movement is required in stretching, it could provide a sufficient alternative or early step in rehabilitation processes to avoid muscle atrophy and performance losses. However, hypertrophic effects were exclusively reported using long-lasting static stretching in the calf muscle. Additionally, when muscles are immobilized in a lengthened position for high-volume stretching, the antagonist muscles are immobilized in a shortened position. Since Williams et al. [76] reported a rapid decrease in serial sarcomeres and considering mechanical stretch as an underlying mechanism of hypertrophy, atrophic effects of the antagonist might be assumed. However, further studies are needed in this area, as well as on the effects in different populations, such as patients.

## Conclusions

To date, the underlying mechanisms of stretch-mediated muscle strength increases and muscle hypertrophy in humans are not fully clarified. Even though some studies using long-lasting stretching of ≥ 30 min per session showed strength increases and hypertrophy, these studies are limited to the calf muscle and opposed by a high number of studies using shorter stretching durations without any effect. Because of high study heterogeneity and limited intensity quantification in stretching studies, further literature seems necessary to state a final conclusion.

Because in humans only a few studies have explored first physiological parameters, the discussion is based on animal results. The scarce evidence from human investigations addressing protein synthesis and muscle damage provides only an inconclusive picture that seems to be in conflict with indications from animal studies. Therefore, the role of mechanical tension in humans needs further clarification by including physiological parameters and intensity quantification. As hypertrophy was exclusively reported in studies using longer stretching durations, extended research addressing underlying mechanisms due to stretching should focus on long stretching durations. Given the different parameters influencing muscle morphology, further factors such as hypoxia, fascial tissue as well as neuronal mechanisms should be included in further research to maximize potential indicated effects.
